# Supplementary material for: Self-charging electrostatic face masks leveraging triboelectrification for prolonged air filtration
Source: Nat Commun. 2022 Dec 20;13:7835. doi: 10.1038/s41467-022-35521-w (PMC9768124; doi:10.1038/s41467-022-35521-w)
Supplement: Supplementary file 1 — Supplementary Information [file 41467_2022_35521_MOESM1_ESM.pdf]

Supplementary Information for

Self-charging electrostatic face masks leveraging triboelectrification for prolonged air filtration

Zehua Peng, Jihong Shi, Xiao Xiao, Ying Hong, Xuemu Li, Weiwei Zhang, Yongliang Cheng, Zuankai Wang, Wen Jung Li, Jun Chen, Michael K.H. Leung, Zhengbao Yang*

*Corresponding author. Email: zb.yang@cityu.edu.hk

Supplementary Information Contents:

Supplementary Figs. 1 to 15
Supplementary Tables 1 to 5
Supplementary Note 1
Supplementary Note 2
Supplementary References (1 to 14)

Other Supplementary Materials for this manuscript include the following:

Supplementary Movie 1

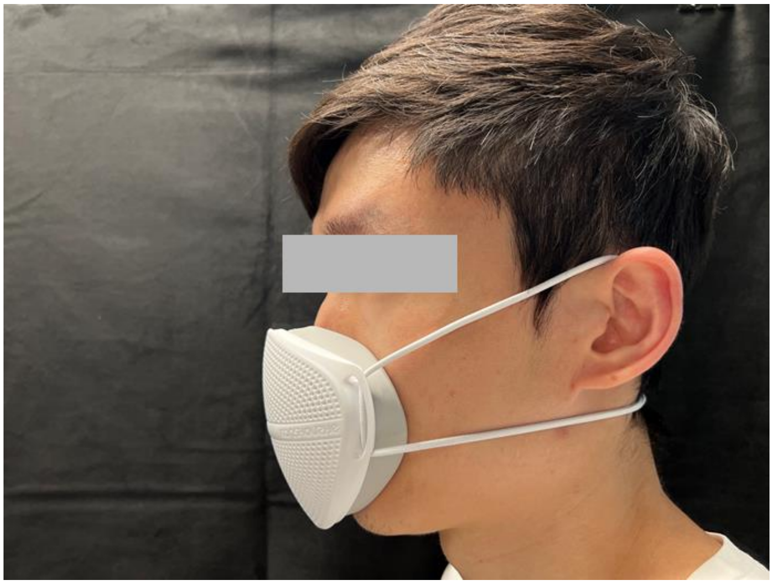

Supplementary Fig. 1. Optical photograph showing a self-charging mask on a person.

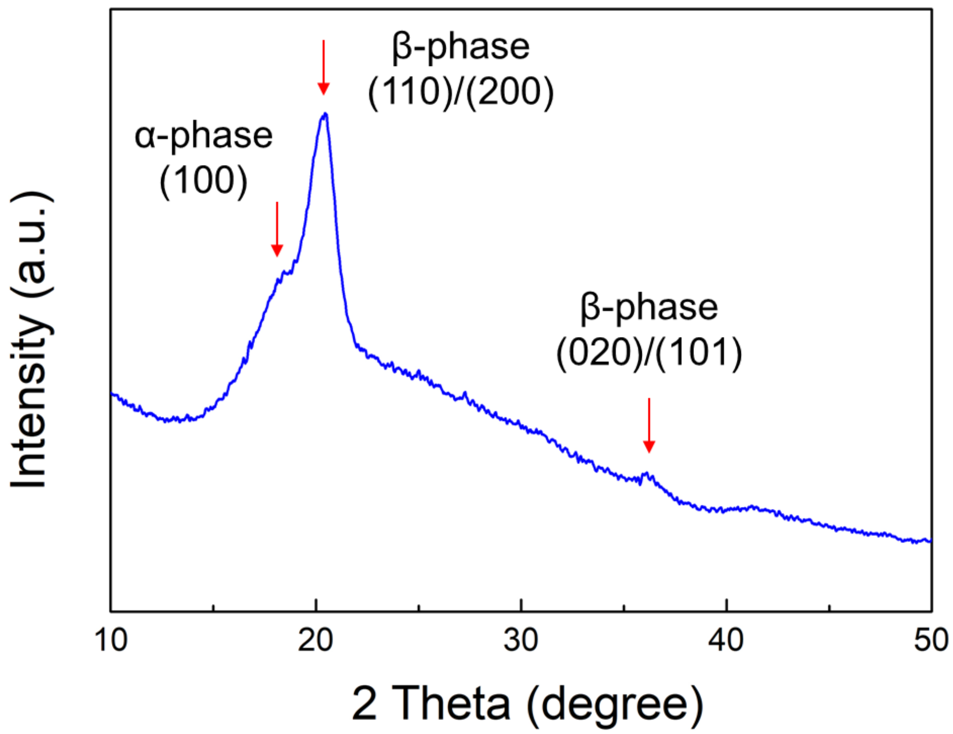

Supplementary Fig. 2. XRD curve showing that the electrospun PVDF film is basically β-phase due to the high voltage polarization in electrospinning.

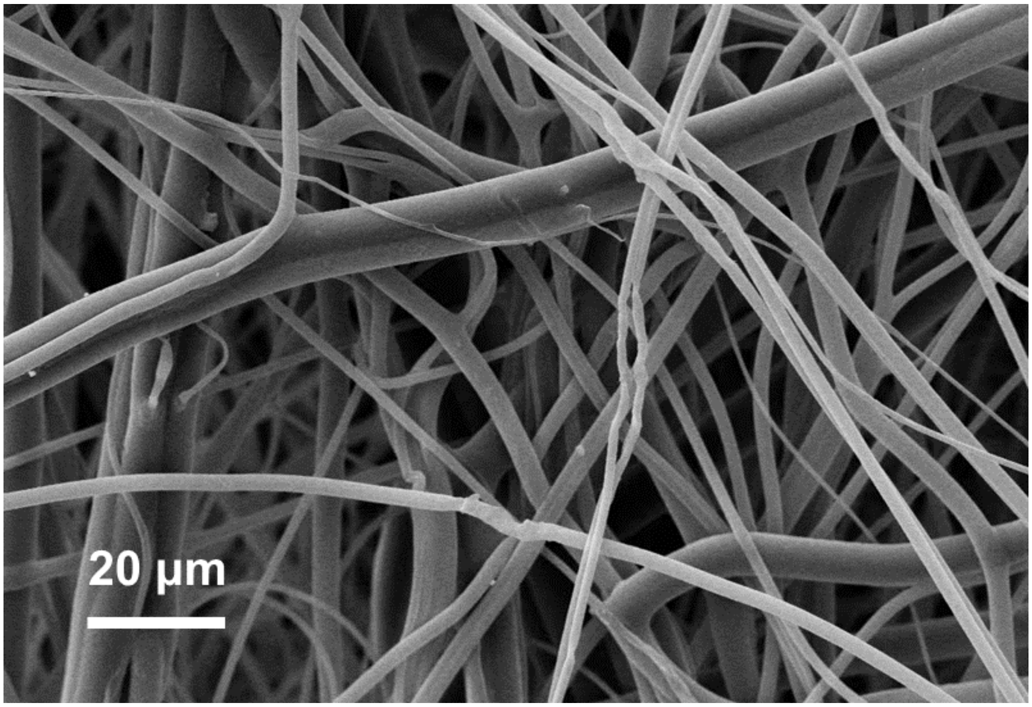

Supplementary Fig. 3. SEM image of the PP melt-brown layer of a surgical mask.

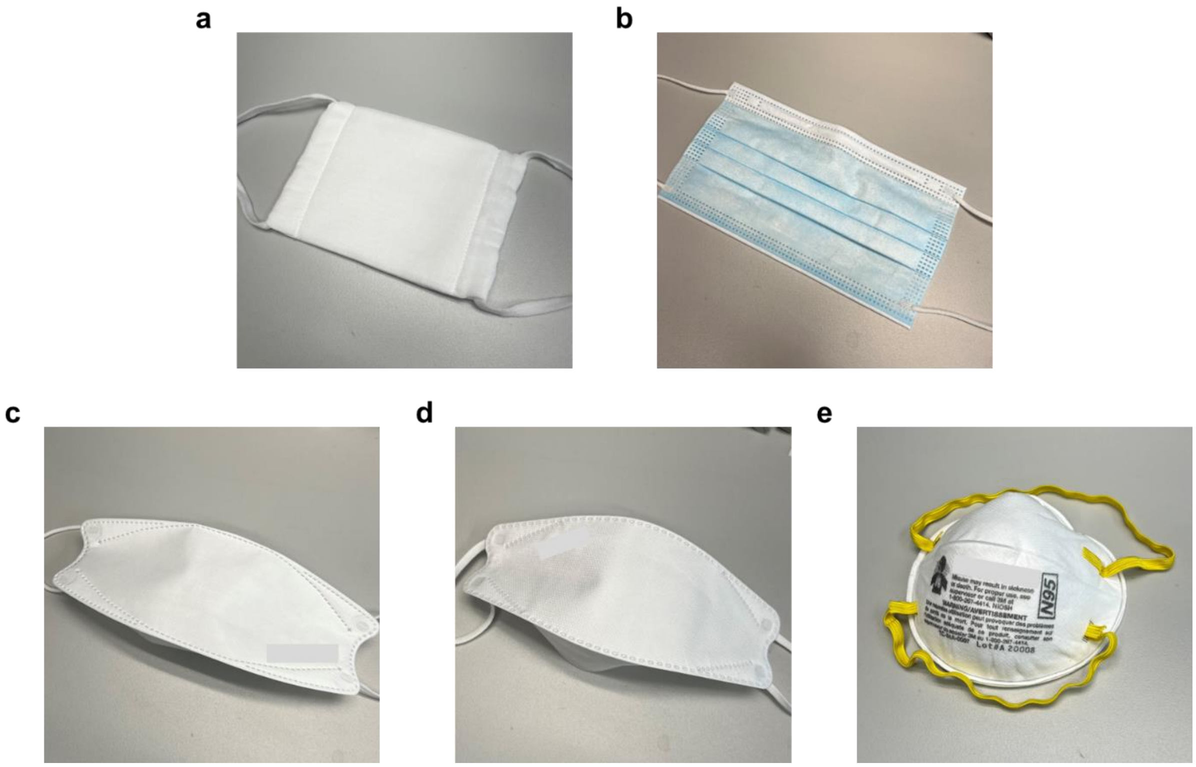

Supplementary Fig. 4. Optical photos of the commercial face masks. **a,** Cotton mask. **b,** Surgical mask. **c,** KF94 respirator. **d,** KN95 respirator. **f,** N95 respirator.

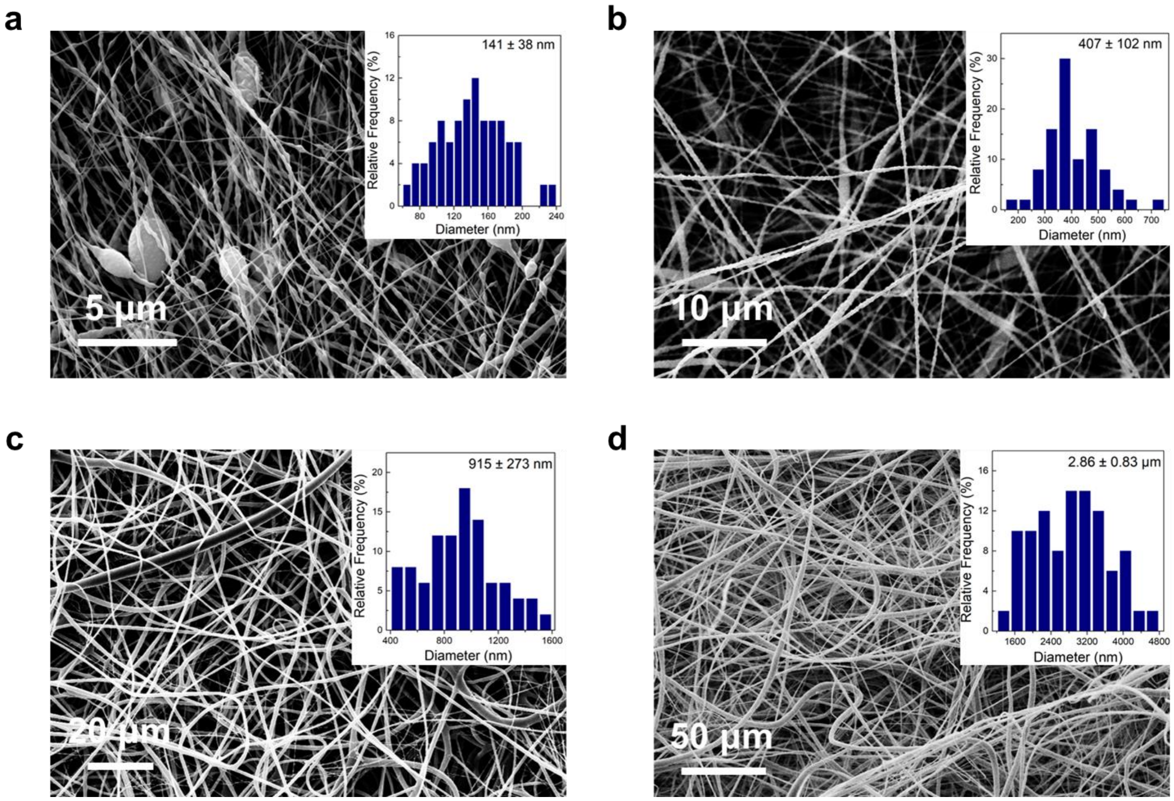

Supplementary Fig. 5. SEM images of electrospun PVDF films with diameters of **a,** 141±38 nm, **b,** 407±102 nm, **c,** 915±273 nm, and **d,** 2.86±0.83 μm. Insets are the fiber diameter distribution.

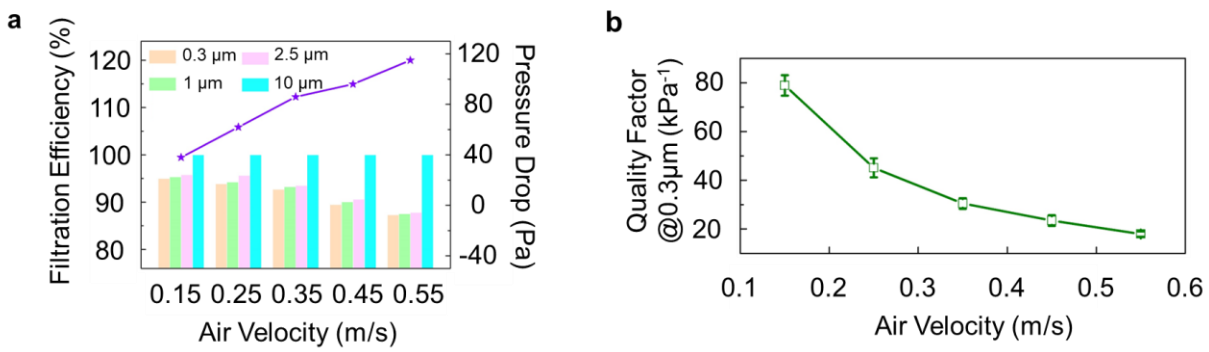

Supplementary Fig. 6. a, Air velocity-dependent filtration efficiency and pressure drop, and **b,** corresponding quality factor. Data are presented as the mean values ± standard deviations (n = 5 independent samples). The five-pointed stars in violet in **a** represent values of the pressure drop.

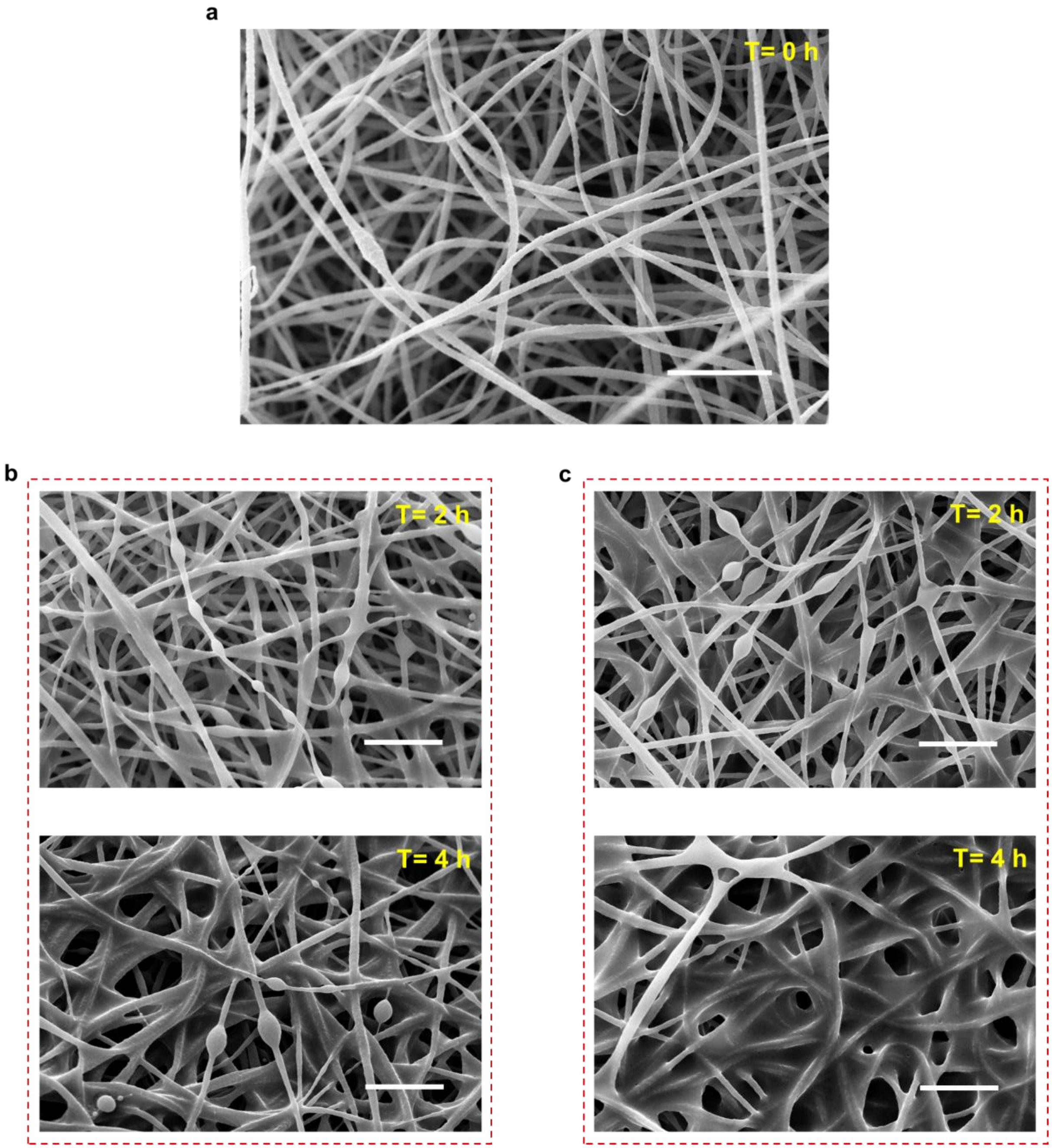

Supplementary Fig. 7. Microscopic characterization revealing the evolution of particle caption. **a,** Before filtration. After filtration **b,** without and **c,** with electrostatic charges. Scale bars: 10 μm.

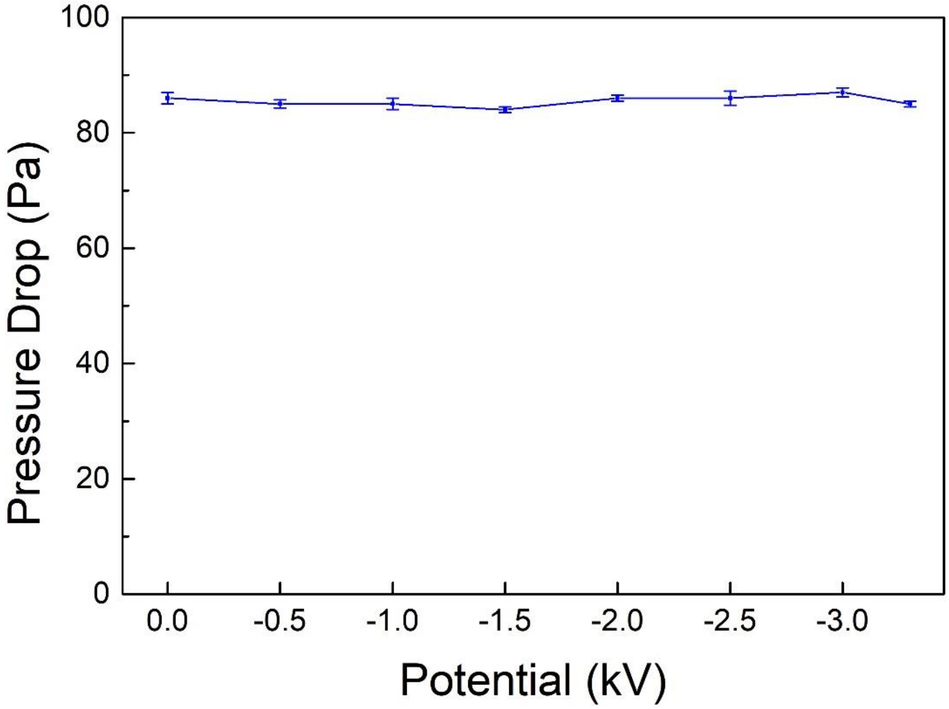

Supplementary Fig. 8. Potential-dependent pressure drop, showing that charge injection exerts no effect on the pressure drop. Data are presented as the mean values ± standard deviations (n = 5 independent samples).

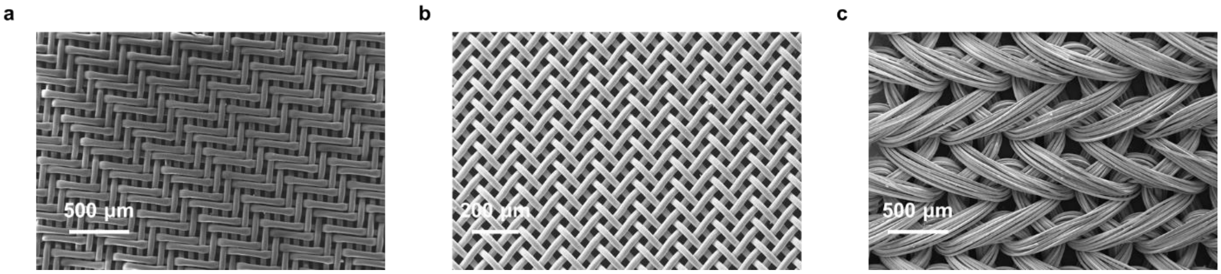

Supplementary Fig. 9. SEM images of the **a,** nylon fabric, **b,** copper mesh, and **c,** conductive fabric.

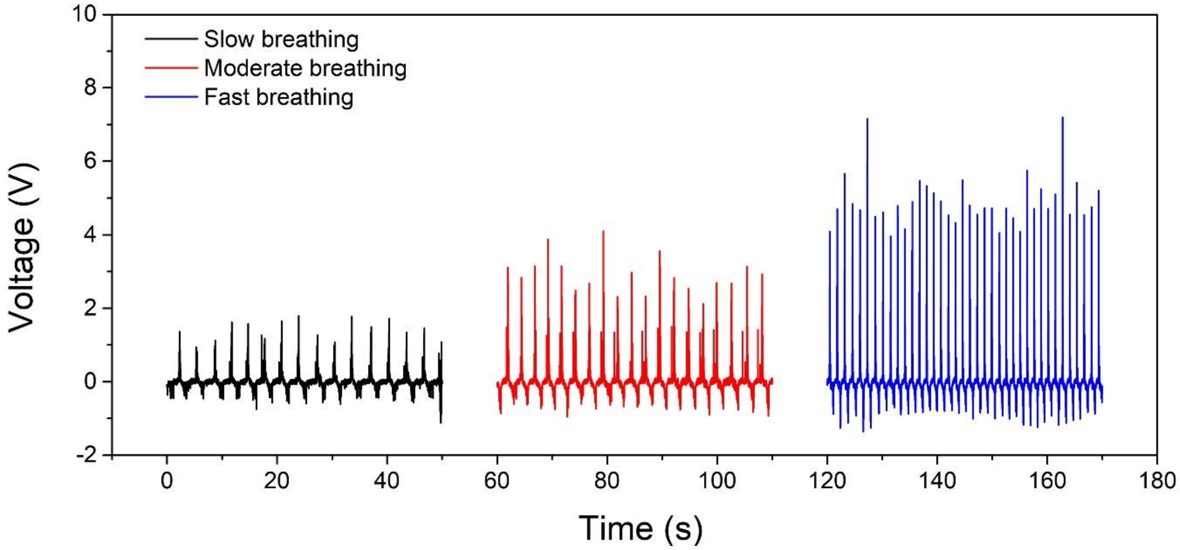

Supplementary Fig. 10. Voltage signals of the two-layer structure filter (PVDF/nylon) under slow breathing, moderate breathing, and fast breathing.

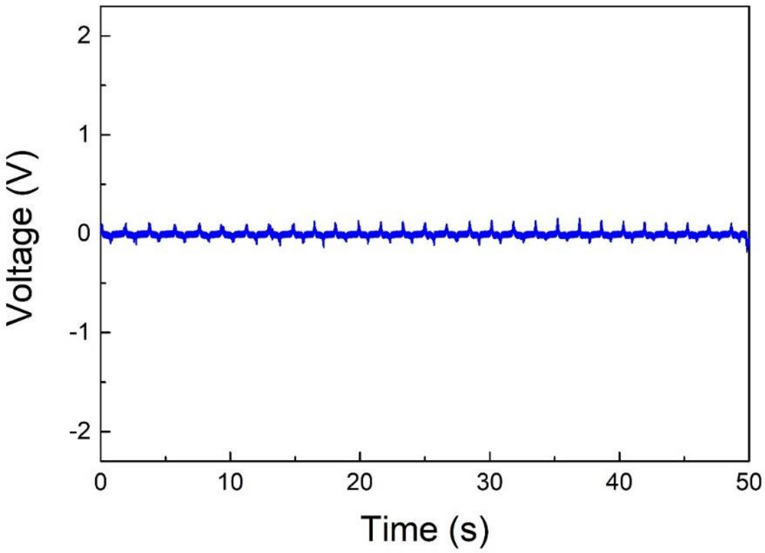

Supplementary Fig. 11. Voltage signal generated with the commercial PP/PP nonwoven pair.

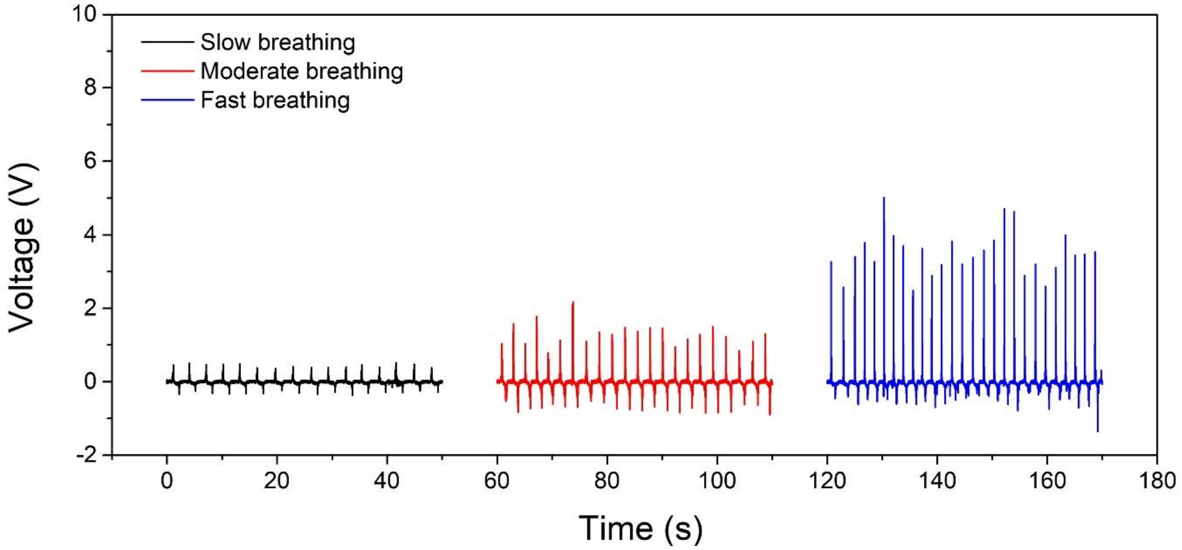

Supplementary Fig. 12. Voltage signals of the copper-PVDF-copper filter under slow breathing, moderate breathing, and fast breathing.

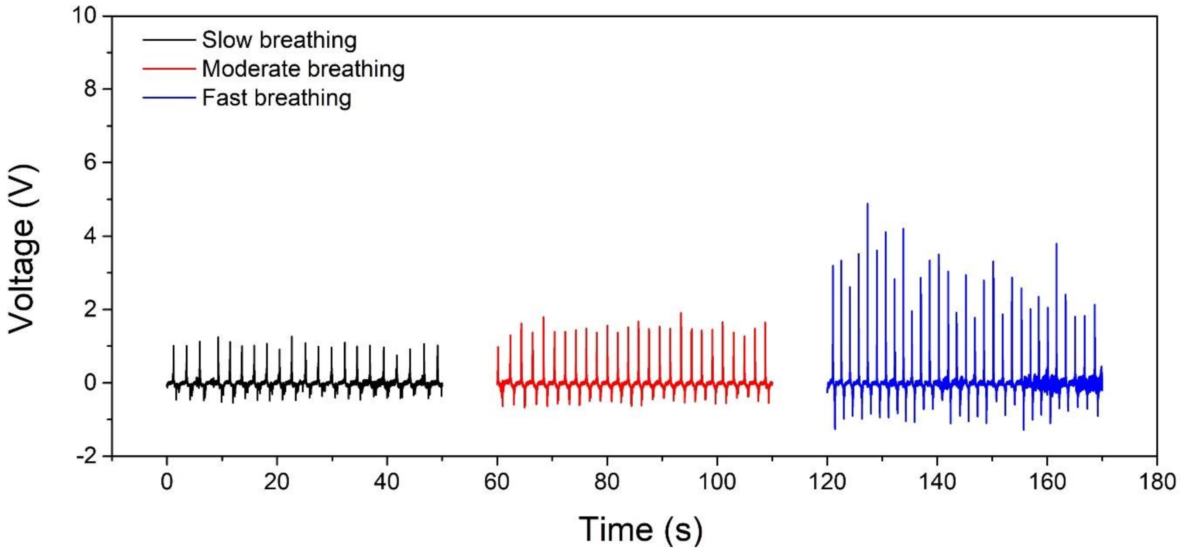

Supplementary Fig. 13. Voltage signals of the conductive fabric-PVDF-conductive fabric filter under slow breathing, moderate breathing, and fast breathing.

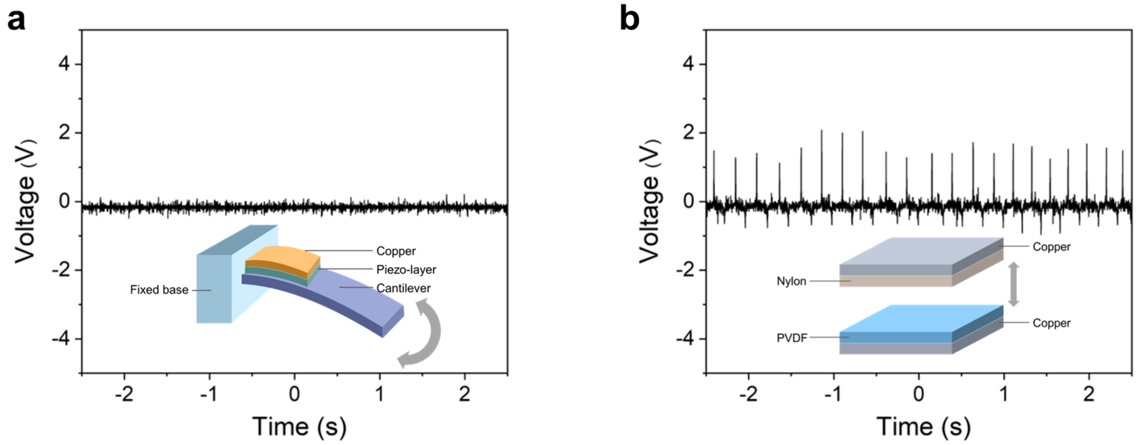

Supplementary Fig. 14. Output voltage signals of the PVDF layer with **a,** piezoelectric and **b,** triboelectric configurations.

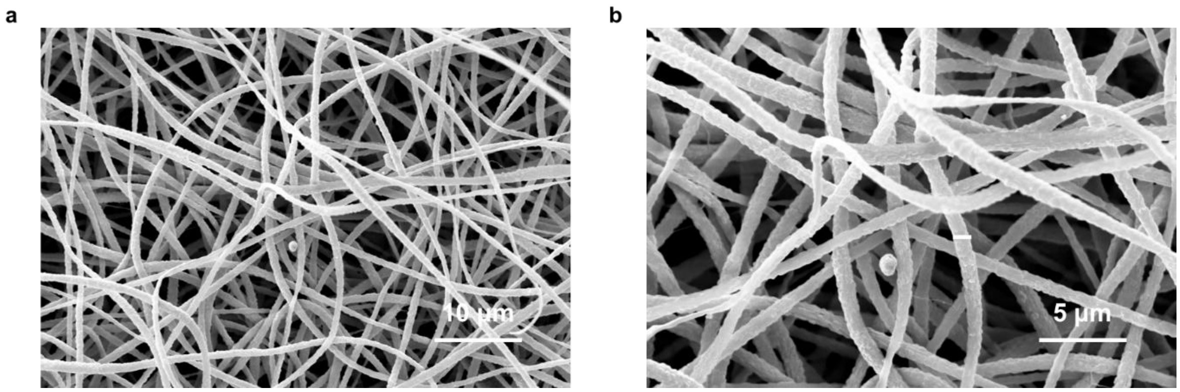

Supplementary Fig. 15. Morphologies of the PVDF fiber after the 60-hour testing. Only a small amount of contaminant was captured, indicating the excellent air quality during the testing days in Hong Kong.

Supplementary Table 1. Cost calculation for the SAF.

	Specification	Price (HKD)	Usage per SAF	Unit Price (HKD)
PVDF powder	120 g	370.4	0.048 g	0.15
DMF	2.5 L	140	0.202 mL	0.01
Acetone	4 L	140	0.304 mL	0.01
Nylon fabric	1 m^2	18.8	160 cm^2	0.3
			Total:	0.47

Supplementary Table 2. Comparison of the SAF with commercial products.

Certification Name for Commercial Masks	Standard	Filtration Efficiency @0.3μm	Pressure Drop (Pa)	Quality Factor (kPa^{-1})	1/Cost	Durability
Surgical mask	ASTM level 2	82.0	54	31.8	3	2
Cotton mask	N/A	24.3	29	9.6	4	1
N95	NIOSH-42 CFR 84	99.9	94	73.5	1	3
KF94	KMOEL-2017-64	97.1	67	53.0	2	3
KN95	GB2626-2006	99.9	71	97.3	3	3
Self-charging air filter (this work)	N/A	98.4	85	48.6	5	5

The Filtration Efficiency @0.3μm, Pressure Drop, and Quality Factor are actual values, while 1/Cost and Durability are evaluated by scores as described in Supplementary Note 1.

Supplementary Table 3. Source data for the performance comparison with previously reported filters.

Filtration efficiency (%)	Pressure drop (Pa)	Challenge particle size	Test air velocity (m/s)	Charging source	Reference
93.77	27	< 1 μm	0.3	Lithium cell/solar panel	*(1)*
94	110	0.3 μm	0.15	TENG with high output voltage*	*(2)*
99.48	9.5	0.3 μm	0.4	High voltage power supply	*(3)*
94	30	0.1 μm	0.053	High voltage power supply	*(4)*
90	18	0.1 μm	0.053	High voltage power supply	*(4)*
86.9	170	0.3 μm	0.3	Self-powered with triboelectrification	*(5)*
94.1	4	53.3 nm	/	TENG with high output voltage	*(6)*
84.7	30	0.3 μm	0.04	Triboelectrification by rubbing	*(7)*
97	17	1 – 2.5 μm	1	TENG with high output voltage	*(8)*
99.6	23	0.3 μm	0.0533	N/A	*(9)*
96.12	133	1 – 2.5 μm	0.21	N/A	*(10)*
98.11	206	1 – 2.5 μm	0.21	N/A	*(10)*
99.64	797.5	0.3 μm	0.7	N/A	*(11)*
94	63	1 μm	0.35	N/A	*(12)*
96.9	112	0.3 μm	0.33	N/A	*(13)*
95	80	0.3 – 10 μm	/	Triboelectrification by rubbing	*(14)*
98.1	84	0.3 μm	0.35	Onsite self-charging	This work

* TENG is short for triboelectric nanogenerator.

Supplementary Table 4. Summary of the electrospinning parameters.

PVDF	Amount (g)	Diameter (nm)	Basis weight (g/m^2)	Thickness (μm)	Solution concentration (wt. %)	Voltage (kV)
Powder	0.2	141	6	17	6.5	15
Powder	0.2	407	6	19	7.5	15
Powder	0.2	694	6	20	10	15
Powder	0.2	915	6	27	10	10
Pellet*	0.2	2863	6	45	10	15
Powder	0.07	694	2	9	10	15
Powder	0.13	694	4	15	10	15
Powder	0.27	694	8	26	10	15
Powder	0.33	694	10	33	10	15

* PVDF pellets (M_w = 530,000, Sigma-Aldrich) were used to fabricate large-diameter fibers.

Supplementary Table 5. Parameters of the corona electret treatment. The measurement was conducted at a temperature of ca. 23 °C and relative humidity of ca. 50%.

Voltage (−kV)	Time (min)	Distance (cm)	Potential (−kV)
7.75	1	2	0.49 ± 0.02*
8.38	3	2	0.99 ± 0.03
9.44	3	2	1.51 ± 0.04
10.61	3	2	1.98 ± 0.06
11.80	3	2	2.52 ± 0.02
13.05	3	2	2.97 ± 0.06
14.4	3	2	3.32 ± 0.06

* The standard deviation was calculated with five independent measurements.

Supplementary Note 1: Qualitative scores of 1/Cost and Durability.

The prices of raw materials used in the SAF are listed in Supplementary Table 1. The prices of the surgical mask, cotton mask, N95, KF94, and KN95 are HK$2.7, HK$0.8, HK$15, HK$6, and HK$3.8, respectively. 1/Cost is scored following the criteria below.

Unit Price (HKD)	<0.5	0.51–1	1.01–5	5.01–10	>10
Rating	5	4	3	2	1

In the radar chart, Durability refers to the stability of electrostatic charges. The self-charging method used in this work can continuously replenish electrostatic charges given there is mechanical oscillation, e.g., breathing, so there is no penalty (5 points). Onsite charging represents that a micro-button battery is integrated into the filter to provide charges in an onsite manner (*1*). This method loses 1 point considering the battery replacement and potential safety issues the battery may cause. Offsite charging methods have been reported in several works (see Supplementary Table 3). An external high-voltage power source (a commercial high-voltage power supply or a triboelectric nanogenerator with a high output voltage) is needed to charge the filter medium. This method loses 2 points as the high-voltage equipment is cumbersome and not portable for use. Both the N95 respirator (and other respirators with similar levels of protection, e.g., KF95, KN95) and the surgical mask undergo an electret treatment in the production. However, such charges decay with time, especially in a humid environment. Hence, they get 2 points. Lastly, other masks, e.g., cotton masks, medical masks, and activated carbon masks, do not experience an electret treatment process and do not even contain a filter medium, so they get 1 point. Complete rating criteria for Durability are listed below.

	Self-charging	Onsite charging	Offsite charging	Electret treatment	Without charging
Rating	5	4	3	2	1

Supplementary Note 2: Fabrication procedures of the piezoelectric device.

We used a cantilever structure to assemble the piezoelectric device. The specific fabrication procedures are as follows. First, the electrospun PVDF films were undergone corona electret treatment (−14.4 kV for 3 min at a distance of 2 cm, resulting in a surface potential of −3.3 kV, as shown in Supplementary Table 5) at room temperature. Second, the as-treated PVDF film (cut into 1.5 cm * 1.5 cm) acted as the piezo-layer and was sandwiched by a top electrode (copper tape; cut into 1.5 cm * 1.5 cm) and a bottom electrode (stainless steel cantilever with a size of 10 cm * 2 cm; piezo-layer was attached on it using a double-side adhesive tape). Third, wires were attached on the two electrodes and connected to an oscilloscope (RTE1024, Rohde & Schwarz) to collect the electrical signals. As shown in Supplementary Fig. 14a, no piezoelectric signals were observed when the cantilever was bent and released. In contrast, triboelectric signals have been observed (Supplementary Fig. 14b) when utilizing a triboelectric configuration (periodic contact separation between PVDF and nylon interfaces).

Supplementary References:

1. Zhang, G.-H. et al. High-performance particulate matter including nanoscale particle removal by a self-powered air filter, *Nat. Commun*. **11**, 1653 (2020).
2. Wang, L. et al. Tribo-charge enhanced hybrid air filter masks for efficient particulate matter capture with greatly extended service life, *Nano Energy* **85**, 106015 (2021).
3. Tian, E. et al. Ultralow resistance two-stage electrostatically assisted air filtration by polydopamine coated PET coarse filter, *Small* **17**, e2102051 (2021).
4. Leung, W. W. F. & Sun, Q. Electrostatic charged nanofiber filter for filtering airborne novel coronavirus (COVID-19) and nano-aerosols, *Sep. Purif. Technol*. **250**, 116886 (2020).
5. Liu, G. et al. Self-powered electrostatic adsorption face mask based on a triboelectric nanogenerator, *ACS Appl. Mater. Interfaces* **10**, 7126–7133 (2018).
6. Gu, G. Q. et al. Triboelectric nanogenerator enhanced multilayered antibacterial nanofiber air filters for efficient removal of ultrafine particulate matter, *Nano Res.* **11**, 4090–4101 (2018).
7. Bai, Y. et al. Washable multilayer triboelectric air filter for efficient particulate matter PM2.5 removal, *Adv. Funct. Mater.* **28**, 1706680 (2018).
8. Gu, G. Q. et al. Triboelectric nanogenerator enhanced nanofiber air filters for efficient particulate matter removal, *ACS Nano* **11**, 6211–6217 (2017).
9. Cheng, Y. et al. Electrospun polyetherimide electret nonwoven for bi-functional smart face mask, *Nano Energy* **34**, 562–569 (2017).
10. Liu, C. et al. Transparent air filter for high-efficiency PM2.5 capture, *Nat. Commun*. **6**, 6205 (2015).
11. He, H. et al. Monitoring multi-respiratory indices via a smart nanofibrous mask filter based on a triboelectric nanogenerator, *Nano Energy* **89**, (2021).
12. Han, K. S. et al. Electrically activated ultrathin PVDF-TrFE air filter for high-efficiency PM1.0 filtration, *Adv. Funct. Mater.* **29**, 1903633 (2019).
13. Wang, N. et al. New hydrogen bonding enhanced polyvinyl alcohol based self-charged medical mask with superior charge retention and moisture resistance performances, *Adv. Funct. Mater.* **31**, 2009172 (2021).
14. Zhang, R. et al. Enhancing the filtration efficiency and wearing time of disposable surgical masks using TENG technology, *Nano Energy* **79**, 105434 (2021).
