# Supplementary material for: Self-charging electrostatic face masks leveraging triboelectrification for prolonged air filtration
Source: Nat Commun. 2022 Dec 20;13:7835. doi: 10.1038/s41467-022-35521-w (PMC9768124; doi:10.1038/s41467-022-35521-w)
Supplement: Supplementary file 3 — Description of Additional Supplementary Files [file 41467_2022_35521_MOESM3_ESM.pdf]

Description of Additional Supplementary Files

File Name: Supplementary Movie 1
Description: Stable electrical signals yielded by the self-charging air-filtering mask under mild breathing
