# Supplementary material for: Self-charging electrostatic face masks leveraging triboelectrification for prolonged air filtration
Source: Nat Commun. 2022 Dec 20;13:7835. doi: 10.1038/s41467-022-35521-w (PMC9768124; doi:10.1038/s41467-022-35521-w)
Supplement: Supplementary file 5 — Reporting Summary [file 41467_2022_35521_MOESM5_ESM.pdf]

nature portfolio

Corresponding author(s): Dr. Zhengbao Yang

Last updated by author(s): Nov 26, 2022

Reporting Summary

Nature Portfolio wishes to improve the reproducibility of the work that we publish. This form provides structure for consistency and transparency in reporting. For further information on Nature Portfolio policies, see our Editorial Policies and the Editorial Policy Checklist.

Statistics

For all statistical analyses, confirm that the following items are present in the figure legend, table legend, main text, or Methods section.

n/a	Confirmed	
☐	☒	The exact sample size (*n*) for each experimental group/condition, given as a discrete number and unit of measurement
☐	☒	A statement on whether measurements were taken from distinct samples or whether the same sample was measured repeatedly
☒	☐	The statistical test(s) used AND whether they are one- or two-sided<br>*Only common tests should be described solely by name; describe more complex techniques in the Methods section.*
☒	☐	A description of all covariates tested
☒	☐	A description of any assumptions or corrections, such as tests of normality and adjustment for multiple comparisons
☐	☒	A full description of the statistical parameters including central tendency (e.g. means) or other basic estimates (e.g. regression coefficient) AND variation (e.g. standard deviation) or associated estimates of uncertainty (e.g. confidence intervals)
☒	☐	For null hypothesis testing, the test statistic (e.g. *F*, *t*, *r*) with confidence intervals, effect sizes, degrees of freedom and *P* value noted<br>*Give P values as exact values whenever suitable.*
☒	☐	For Bayesian analysis, information on the choice of priors and Markov chain Monte Carlo settings
☒	☐	For hierarchical and complex designs, identification of the appropriate level for tests and full reporting of outcomes
☒	☐	Estimates of effect sizes (e.g. Cohen's *d*, Pearson's *r*), indicating how they were calculated

Our web collection on statistics for biologists contains articles on many of the points above.

Software and code

Policy information about availability of computer code

Data collection	No software was used.
Data analysis	No software was used.

For manuscripts utilizing custom algorithms or software that are central to the research but not yet described in published literature, software must be made available to editors and reviewers. We strongly encourage code deposition in a community repository (e.g. GitHub). See the Nature Portfolio guidelines for submitting code & software for further information.

Data

Policy information about availability of data

All manuscripts must include a data availability statement. This statement should provide the following information, where applicable:

- Accession codes, unique identifiers, or web links for publicly available datasets
- A description of any restrictions on data availability
- For clinical datasets or third party data, please ensure that the statement adheres to our policy

The data that support the findings detailed in this study are available in the Article and its Supplementary Information or from the corresponding authors upon reasonable request.

Human research participants

Policy information about studies involving human research participants and Sex and Gender in Research.

Reporting on sex and gender	In this study, we invited two females and two males to wear our self-charging mask to evaluate its durability of filtration efficacy. The duration test lasted for 60 hours in total, including 30 hours of wearing and 30 hours of resting. No gender-based analyses have been performed since face masks are protective products regardless of the gender and commercial mask products also do not provide a gender-related parameter report.
Population characteristics	The only population characteristics of the human research participants is the age. At the time of the test, two female participants were 22 and 26 years old, while two male participants were 28 and 32 years old.
Recruitment	All the participants are labmates of the authors. There are no self-selection bias for the participant recruitment.
Ethics oversight	The Institutional Review Board at the City University of Hong Kong approved the study protocol

Note that full information on the approval of the study protocol must also be provided in the manuscript.

Field-specific reporting

Please select the one below that is the best fit for your research. If you are not sure, read the appropriate sections before making your selection.

☒ Life sciences ☐ Behavioural & social sciences ☐ Ecological, evolutionary & environmental sciences

For a reference copy of the document with all sections, see nature.com/documents/nr-reporting-summary-flat.pdf

Life sciences study design

All studies must disclose on these points even when the disclosure is negative.

Sample size	No sample-size calculation was performed. This study focuses on the self-charging air filter (SAF). To assemble a whole mask, the SAF was integrated into a commercial mask shell. Therefore, the sample (each layer of the SAF) size complies with the size of the mask shell, which has an area of approximate 60 cm2.
Data exclusions	No data were excluded from the analyses.
Replication	We built a standard testing platform to evaluate the filtration performance of the mask. Thanks to this platform, the data on filtration efficiency and pressure drop were replicable. For other experimental findings, the reproducibility was good since all the instruments and experimental methods were fixed.
Randomization	There was no deliberate allocation, considering the face masks are protective products for all human beings and the parameter report of the commercial mask products does not consider the individual difference.
Blinding	The authors were not blinded to group allocation during data collection. The reason lies in that face masks are protective products for all human beings and the parameter report of the commercial mask products does not consider the individual difference, thereby blinding was not relevant to this study.

Reporting for specific materials, systems and methods

We require information from authors about some types of materials, experimental systems and methods used in many studies. Here, indicate whether each material, system or method listed is relevant to your study. If you are not sure if a list item applies to your research, read the appropriate section before selecting a response.

Materials & experimental systems

n/a	Involved in the study	
☒	☐	Antibodies
☒	☐	Eukaryotic cell lines
☒	☐	Palaeontology and archaeology
☒	☐	Animals and other organisms
☒	☐	Clinical data
☒	☐	Dual use research of concern

Methods

n/a	Involved in the study	
☒	☐	ChIP-seq
☒	☐	Flow cytometry
☒	☐	MRI-based neuroimaging
